# Leaky Gut and the Ingredients That Help Treat It: A Review

**DOI:** 10.3390/molecules28020619

**Published:** 2023-01-07

**Authors:** Ricardo Santos Aleman, Marvin Moncada, Kayanush J. Aryana

**Affiliations:** 1 School of Nutrition and Food Sciences, Louisiana State University Agricultural Center, Baton Rouge, LA 28081, USA; 2 Department of Food, Bioprocessing & Nutrition Sciences and the Plants for Human Health Institute, North Carolina State University, North Carolina Research Campus, Kannapolis, NC 27599, USA

**Keywords:** leaky gut, ingredients, health

## Abstract

The human body is in daily contact with potentially toxic and infectious substances in the gastrointestinal tract (GIT). The GIT has the most significant load of antigens. The GIT can protect the intestinal integrity by allowing the passage of beneficial agents and blocking the path of harmful substances. Under normal conditions, a healthy intestinal barrier prevents toxic elements from entering the blood stream. However, factors such as stress, an unhealthy diet, excessive alcohol, antibiotics, and drug consumption can compromise the composition of the intestinal microbiota and the homeostasis of the intestinal barrier function of the intestine, leading to increased intestinal permeability. Intestinal hyperpermeability can allow the entry of harmful agents through the junctions of the intestinal epithelium, which pass into the bloodstream and affect various organs and systems. Thus, leaky gut syndrome and intestinal barrier dysfunction are associated with intestinal diseases, such as inflammatory bowel disease and irritable bowel syndrome, as well as extra-intestinal diseases, including heart diseases, obesity, type 1 diabetes mellitus, and celiac disease. Given the relationship between intestinal permeability and numerous conditions, it is convenient to seek an excellent strategy to avoid or reduce the increase in intestinal permeability. The impact of dietary nutrients on barrier function can be crucial for designing new strategies for patients with the pathogenesis of leaky gut-related diseases associated with epithelial barrier dysfunctions. In this review article, the role of functional ingredients is suggested as mediators of leaky gut-related disorders.

## 1. Introduction

**Figure 1:**
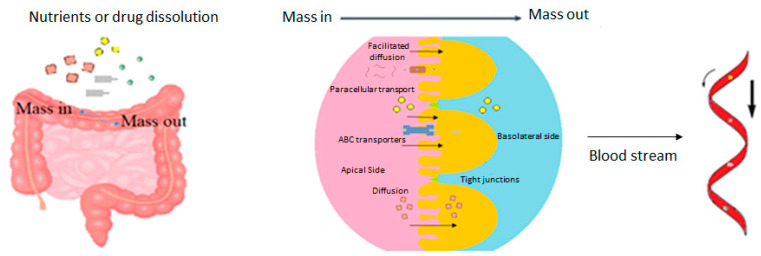
Representation of the structure of the intestinal membrane showing the different routes of drug and nutrient transport.

**Figure 2:**
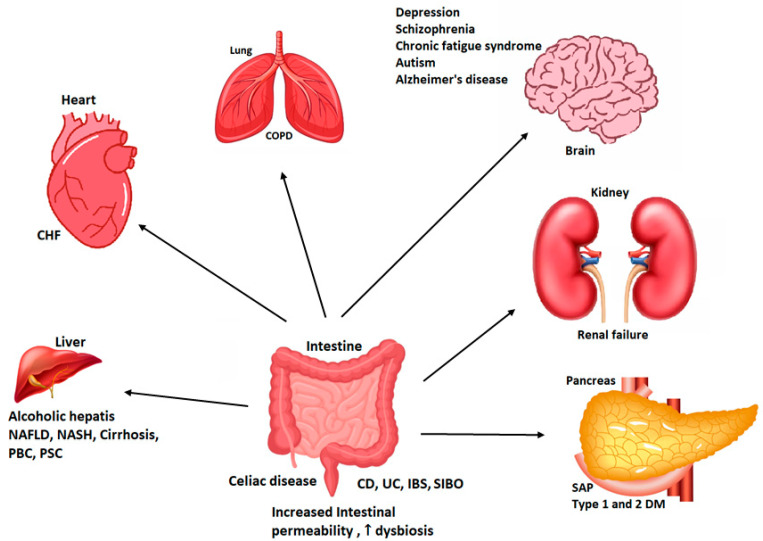
Relationship between leaky gut syndrome and intestinal dysbiosis with various diseases. NAFLD: nonalcoholic fatty liver disease; NASH: nonalcoholic steatohepatitis; PBC: primary biliary cholangitis; SAP: severe acute pancreatitis; DM: diabetes mellitus; SIBO: small intestinal bacterial overgrowth; COPD: chronic obstructive pulmonary disease; CHF: congestive heart failure; CD: Crohn’s disease; UC: ulcerative colitis (ulcerative colitis); IBS: inflammatory bowel diseases.

The human body is exposed daily to potentially harmful substances and agents. These infectious agents can upset the balance between health and disease. The gastrointestinal tract transports water and electrolytes and secretes water and protein towards the intestinal lumen. This action has a defense function that prevents harmful substances from entering the intestinal barrier [1] The intestinal barrier forms two complex layers, which consist of an apical barrier and basolateral barrier (Figure 1). The small intestine mixes food with digestive juices from the pancreas, liver, and intestine and pushes the mixture forward to continue the digestion process. The cellular walls of the small intestine absorb digested nutrients and drugs through diffusion, ATP-binding cassette (ABC) transporters and paracellular transportation into the bloodstream (Figure 1). The interaction of both barriers allows the maintenance and balance of intestinal homeostasis [2], which is capable of discriminating between commensal microorganisms (beneficial for the host), pathogens, nutrients and inflammatory particles [3]. Under normal conditions, an intact intestinal barrier prevents the transmission of pathogens, pro-inflammatory substances and antigens to the internal environment. However, a lack of intestinal integrity favors its entry and could trigger a disease or inflammation [4]. Dysfunction of the intestinal epithelial barrier and increased permeability results in a “leaky gut” that is associated with intestinal disorders such as inflammatory bowel disease (IBD), bowel syndrome irritable liver disease (ILD), alcoholic liver disease, nonalcoholic fatty liver disease, steatohepatitis, liver cirrhosis, and collagen diseases (Figure 2). Leaky gut is also related to diseases that are not intestinal disorders such as diabetes mellitus, among others, represented in Figure 2.

Intestinal permeability is defined as the unmediated passage through the intestinal epithelium of medium-sized hydrophilic molecules that occurs down a gradient of concentration [5,6]. An increase in intestinal permeability is a sign of a disturbed intestinal barrier [7]. According to the leaky gut syndrome (LGS) hypothesis, intestinal hyperpermeability may allow the entry of harmful microorganisms, toxins, or undigested food particles through the junctions of the intestinal epithelium, reaching the bloodstream and being able to affect the hormonal, immune, nervous, respiratory or reproductive systems [8]. In fact, an increase in the permeability of the intestine due to changes in the functioning and/or in the expression levels of the TJ proteins cause leaky gut syndrome or LGS.

The incidence of inflammatory bowel and leaky gut diseases is on the rise in countries that adopt a Western lifestyle. Its pathogenesis is not well defined, but it is associated with multifactorial causes. However, in genetically predisposed individuals, different environmental factors trigger alterations in the immune response; as a result, tolerance towards the commensal intestinal microbiota is lost, causing tissue damage and chronic inflammation. Among the environmental risk factors is diet. Diets high in sucrose, refined carbohydrates, polyunsaturated fatty acids, and omega-6 and low in fiber are associated with an increased risk of presenting these intestinal disorders [9]. Nutritional recommendations for its control cannot be generalized since all patients do not respond in the same way. The emergence of disciplines such as nutrigenetics, nutrigenomics and epigenetics allows a greater understanding of the pathogenesis of this disease and, in turn, opens the possibility of an individualized approach from a nutritional point of view. The research on treating the intestinal permeability is mostly based on avoidance of high amounts of sugar and fat and implementation of FODMAP (fermentable oligosaccharides, disaccharides, monosaccharides, and polyols), prebiotics, probiotics, fibers, glutamine, short-chain fatty acids, quercetin, and metformin [2]. There are some functional foods and ingredients that have shown great potential in treating leaky gut. Some herbs, polyphenols, amino acids, minerals, antioxidants, and food products could increase functionality against intestinal permeability. Therefore, this review article aims to explore foods and ingredients that can help prevent or treat leaky gut syndrome.

## 2. Components of the Intestinal Barrier

**Figure 3:**
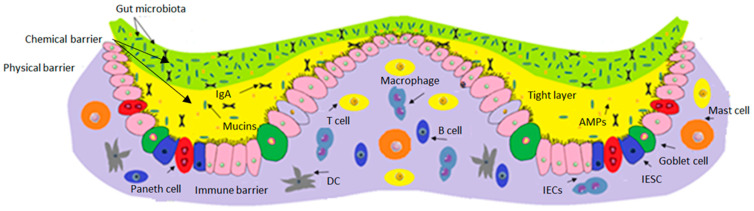
Composition and anatomical structure of the gut barrier.

The composition and anatomical structure of the gut barrier is shown in Figure 3. From top to bottom, there is a microbiota barrier, a chemical barrier, a physical barrier, and an immune barrier. The chemical barrier contains microorganisms, immunoglobulin A (Ig A), mucins, AMPs (adenosine monophosphates) and antibacterial peptides. The physical barrier comprises intestinal epithelial cells (IECs), goblet cells (synthesis and release of mucins), Paneth cells (synthetic AMPs), and intestinal stem cells. The immune barrier is mainly consists of T cells, B cells, macrophages, dendritic cells (DC), and mast cells.

The first line of defense is found in the gastrointestinal tract and the intestinal lumen. The intestinal barrier constitutes the interface between the external and internal environment [10]. It comprises various physical, cellular, and chemical components that contribute to immunological functions (Figure 3) [11]. The inhibition of pathogenic microorganisms and antigens is produced by the action of gastric, pancreatic, and bile secretions. Digestive enzymes, including proteases, lipases, amylases, and nucleases, act as a barrier to those pathogens that could come from the diet [12]. It is desired to have a microflora that competes with pathogens for nutrients, metabolizing proteins and carbohydrates, and synthesizing vitamins [13]. The beneficial microflora can also produce many metabolic products that moderate the interaction between the epithelium and the immune system and generate antimicrobial substances, inhibiting pathogens [14].

Secondly, it is essential to consider the microflora from the mucus. The mucus varies throughout the intestine. In the small intestine, the mucus forms a thin, discontinuous layer, which facilitates the absorption of nutrients, while in the large intestine, it has two layers. The two layers consist of an inner layer, in which there are no bacteria, and a layer of external mucus that physically separates the intestinal lumen from the epithelium. These layers limit the entry of the microbiota to the apical side of the epithelium and provide protection [3]. The mucus components are water (more than 98%), mucins, and glycoproteins. More than 18 mucin-type glycoproteins have been identified [1]. The glycocalyx (carbohydrate-rich layer that covers the mucosal epithelial cells) from the inner layer and MUC2 from the outer layer is essential for disease prevention [11]. In addition, mucus contains secretory immunoglobulin A, antimicrobial products, peptides trefoil (trefoil factor family, TFF), cathelicidins, and ribonucleases, which are responsible for reinforcing the physical separation of the microbiota, forming a gradient from the epithelium to the lumen [11].

Thirdly, the intestinal epithelium, with its tight junctions, is the essential component of the intestinal barrier and separates the microbiota from underlying immune cells forming an epithelial barrier [15]. The epithelial barrier is composed of a monolayer of specialized and polarized epithelial cells renewed every 3 to 5 days. The crypts contain the pluripotent stem cells that continually divide and differentiate themselves as they emigrate towards the tip of the villus, generating the different cell types of the epithelium (Figure 3)—enterocytes, Goblet cells, enteroendocrine cells, and M cells—or remain in the crypts: Paneth cells [16]. The epithelial cells are capable of phagocytizing bacteria and can neutralize bacterial toxins [17]. The epithelial cells also contain the recognition receptor patterns (RRPs) and pathogen-associated molecular patterns (PAMs) that must be subject to stringent controls to avoid inappropriate immune stimulation and inflammation. RRPs include Toll-like receptors (TLRs) and proteins with nucleotide oligomerization domains (NODs) [18]. These defense mechanisms are stimulated and activated when increasing the secretion of peptides, antimicrobials, cytokines, and chemokines [19].

Beneath the intestinal epithelium resides the lamina propria that contains innate and adaptive immune cells, including macrophages, regulatory T cells, B cells, neutrophils, dendritic cells, plasma cells, and mast cells, protecting against microorganisms that penetrate the epithelium. Intraepithelial T lymphocytes and dendritic cells form a network under the epithelium and emit processes between epithelial cells, to which they bind via tight junctions to maintain the epithelial seal (Figure 3) [10].

## 3. Intercellular Junctions of the Intestinal Epithelium

**Figure 4:**
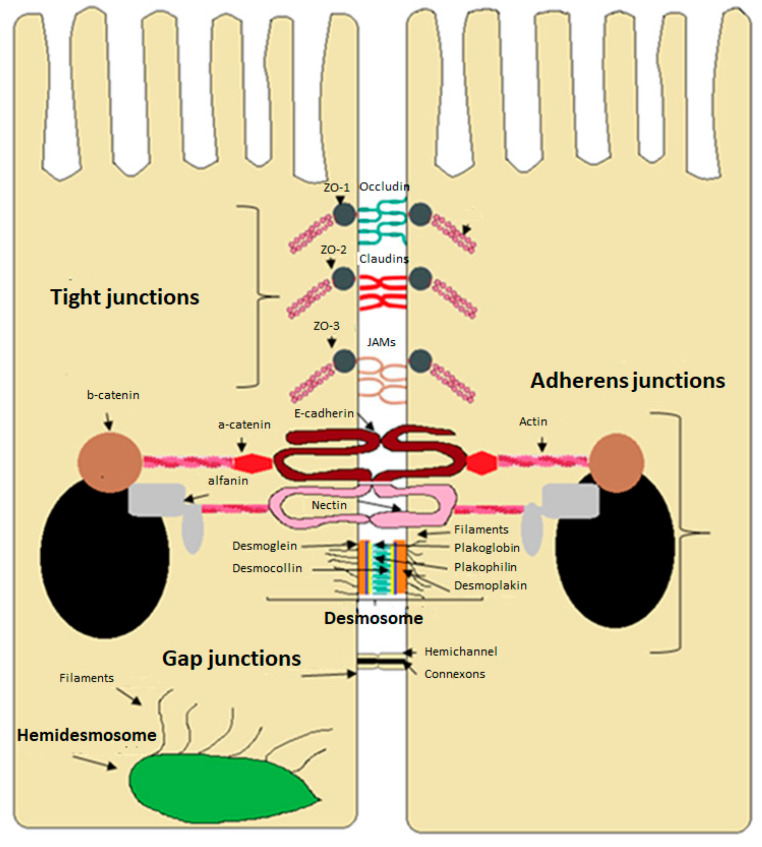
Intercellular junctions of the intestinal epithelium.

For cells to form an epithelium, they need to be attached to the membrane by intercellular junctions. These junctions are classified into three groups (Figure 4): intercellular junctions, anchor junctions, and communicating junctions [20,21]. Tight junction proteins (TJs) are generated by assembling multiple proteins located in the apical part of the epithelium between neighboring cells and control the permeability of the transport pathway paracellular, restricting the passage of ions and solutes. In addition, they maintain the polarity of the epithelial cells by preventing the passage of molecules (lipids and proteins) from the apical membrane to the basolateral and vice versa, so the TJs have a very important function in the establishment of the intestinal barrier. They consist of integral transmembrane proteins that include occludin, tricellulin, claudins, and junctional adhesion molecules (JAMs) and peripheral proteins known as zonula occludens (ZO-1, ZO-2, ZO-3), which bind to actin filaments (Figure 4) [22,23]. Tight junction proteins are highly regulated, which is critical for maintaining the integrity of the normal barrier. The epithelial cells of the intestine proliferate and renew rapidly, and the tight junction proteins must be regulated to avoid any deleterious effect on the integrity of the barrier. The tight junction proteins can adapt to the different demands of the cells, sealing, opening, and maintaining paracellular transport under different physiological and pathological conditions [24]. Regulation is complex and performed by multiple proteins and signaling pathways, such as protein kinases C, A, and G (PKC, PKA, and PKG), phosphatase, myosin light-chain kinase (MLCK), mitogen-activated protein kinases (MAPK), and the phosphatidylinositol-3 kinase pathway (B/Akt, PI3K/Akt). Phosphorylation of occludin is responsible for opening and sealing tight junctions [3]. The plasticity of TJs is essential for gastrointestinal functions, epithelial renewal, and morphogenesis. Under normal physiological conditions, tolerance and homeostasis are maintained with intestinal permeability controlled. However, any defects in the barrier with the TJs open in a deregulated and prolonged way can allow the passage of antigens from the diet or bacteria, a situation known as leaky gut syndrome [25].

Adherent junctions regulate adhesion between adjacent cells through transmembrane adhesion molecules of the catenins and protein complexes associated with the actin cytoskeleton. The AJs are located on the lateral membrane below the TJs and are necessary to assemble and maintain tight joints. E-cadherin is among the isoforms of cadherin in epithelial tissues and participates in cellular processes, cell proliferation, the establishment of cell polarity, and remodeling of the actin cytoskeleton [26]. Desmosomes are intercellular junctions composed of desmocolins, desmoglein, and cadherins. These intercellular junctions can also act as intracellular signaling mediums [26]. Gap junctions are made up of six transmembrane proteins called connexins. They allow communication between cells, performing an essential function in the development, growth and differentiation of epithelial cells [12].

## 4. Gut Microbiome and Leaky Gut

The gut has more than 100 trillion bacteria [27], with an aggregate biomass of approximately 1.5 kg [28], composed of more than 200 microbial strains in an individual and more than 90% of the dominant bacterial species belonging to the phylum *Firmicutes* and *Bacteroidetes* [29,30]. The genome has between 20,000 and 25,000 protein-coding genes, while the genome of the bacterial community in the human intestine is approximately 9 million genes [31], capable of providing characteristics that the human genome does not possess. Some of the observations made by investigations of the intestinal microbiota in adults are a low amount of *Firmicutes* and an abundance of *Bacteroidetes* [32]. Regarding the composition of the intestinal microbiota, it is made up of bacteria, viruses, fungi, and protozoa, although mainly bacteria. The microflora that populates the gut is, for the most part, anaerobes, although we can also find aerobes. The main bacterial species that inhabit the tract are those of the phylum *Bacteroidetes* (*Prevotella, Porphyromonas*), *Firmicutes* (*Clostridium, Eubacteria*), and *Actinobacteria* (*Bifidobacterium*). Other species we can find are those of the *Lactobacillus*, *Streptococcus*, and *Escherichia coli*, although to a lesser extent [32]. The microbial community that harbors the gastrointestinal tract is diverse and host specific. 

The implications of the microbiota in the health of individuals are very numerous, from the stimulation of the immune system [10,33,34], the degradation of dietary fibers, the increase in function and motility of the gastrointestinal tract facilitate the absorption of nutrients and the inhibition of pathogens. The gut contributes to the maintenance of the defense and repairs the gastrointestinal mucosa [33]. Through protein degradation and a reduction in sulfur compounds, it generates hydrogen sulfide, which has antihypertensive effects [35] and seems to have overlapping actions with nitric oxide and prostaglandins, exerting many anti-inflammatory and antihypertensive effects [36].

Bacterial metabolism also degrades plant polysaccharides and generates short-chain fatty acids that represent ≥10% of the calories absorbed daily by an individual [28]. In addition, the microbiota produces other beneficial metabolites such as polyamines [37] and vitamins (B and K). However, it can also produce harmful metabolites such as ammonia from urea and uremic toxins such as trimethylamine N-oxide (TMAO), p-cresyl sulfate (PCS), and indoxyl sulfate (IS), which cause systemic inflammation and other significant effects, such as alterations in the metabolism of drugs due to the inhibition of some isoenzymes, highlighting CYP3A4, which are extremely important in the metabolism of approximately 40% of drugs and would partly explain the variability between individuals with kidney failure [38].

The intestinal microflora is regarded as crucial for the health status of the host. In homeostasis, the relationship between the host and the microbiota is mutualistic. However, a breakdown of this balance, known as dysbiosis, could contribute to the development of the disease [39]. The gut microbiota has a crucial function in the energy and metabolic regulation of the human being since it provides up to 10% of our calories consumed daily [39]. Through the fermentation of food, the microbiota releases metabolites and short-chain fatty acids, which have anti-inflammatory properties and contribute to intestinal homeostasis [40]. The microbiota is considered, together with environmental, genetic, and immunological factors [41], an essential element in the development of inflammatory bowel disease, either as a mechanism that predisposes or protects against the development of intestinal inflammation [29,42]. To sum up, the gut microbiota performs several functions, such as regulating various nutrients and regulating the immune system, which can prevent and treat intestinal inflammation [43].

## 5. Diseases Related to the Alteration of Intestinal Permeability

### 5.1. Inflammatory Bowel Disease

Inflammatory bowel disease (IBD) involves several chronic remitting diseases, of which Crohn’s disease (CD) and ulcerative colitis (UC) are probably the most common. The two differ mainly in the area of the intestine they affect: the first can appear throughout the gastrointestinal tract, although it mainly affects the ileum and cecum, and the second appears mainly in the colon and rectum [44]. Although the etiology of IBD is unknown, a high level of intestinal inflammation is associated with an alteration of the tight junctions. In addition, it has been observed that patients have higher intestinal permeability than healthy subjects [21]. On the one hand, active UC is associated with a decrease in claudin-1, claudin-4, claudin-7, and occludin and an increase in claudin-2. The CD is associated with decreased claudin-3, claudin-5, and claudin-8, as well as increased expression of claudin-2 [45]. For all these reasons, barrier dysfunction in patients with these diseases is related to inflammatory responses and TJ alteration [23].

### 5.2. Irritable Bowel Syndrome and Other Intestinal Disorders

Irritable bowel syndrome (IBS) is a functional digestive disorder characterized by frequent abdominal pain related to shifts in the frequency and formation of bowel actions [44]. Intestinal permeability has also been associated with the pathogenesis of IBS. Specifically, IBS patients showed lower levels of the protein zonula occludens (ZO)-1 and occludin in intestinal tissue. They studied the production of cytokines in peripheral blood mononuclear cells PBMCs), and these patients (most notably those with diarrhea) showed elevated basal levels of TNF-α, IL-1β, and IL-6 in serum [4].

As we have already indicated, various diseases have been related to dysbiosis of the intestinal microbiota, microbial translocation, and dysfunction of the intestine’s barrier function. Among them, we can highlight obesity, chronic heart failure, Alzheimer’s disease, cancer, diabetes, and autoimmune diseases. The function of our immune system is to defend ourselves against infections and other diseases. However, in immune disorders, our body becomes the aggressor and attacks the body’s cells, causing damage [46]. Type 1 diabetes and celiac disease are examples of autoimmune diseases that are discussed further.

### 5.3. Obesity

Obesity is a chronic illness distinguished by an overabundance of adipose tissue in the body. According to the WHO (World Health Organization), obesity is defined when the BMI (Body Mass Index) is equal to or greater than 30 kg/m^2^ [47]. Obesity has been associated with increased intestinal permeability. In genetically obese mouse models, there is an increase in intestinal permeability and plasma endotoxins and proinflammatory cytokines, such as interleukin one beta (IL-1β), interleukin-6 (IL-6), interferon-gamma (INFγ), and tumor necrosis factor (TNF-α), compared to wild-type mice. On the other hand, obesity induced by a high-fat diet (diet-induced obesity, DIO) is linked to changes in the population of intestinal bacteria related to inflammation and increased intestinal permeability due to the reduction in gene expression linked to TJs, including ZO-1 and occludin. All this indicates that obesity-induced inflammation may be associated with changes in the integrity of the tight junctions and the intestinal microbiota [48,49].

### 5.4. NASH and NAFLD

Nonalcoholic fatty liver disease (NAFLD) is a liver disease caused by excessive accumulation of fats within liver cells, not primarily caused by alcohol consumption. On the other hand, in nonalcoholic steatohepatitis (NASH), the patient, in addition to fat, can present other alterations in the liver, such as inflammation and scars [50]. Changes in the gut microbiota composition in NAFLD patients increased LPS in circulating plasma, subsequently triggering inflammation [51]. These plasma LPS and proinflammatory cytokines simultaneously increase intestinal permeability [51]. The increased permeability in NAFLD patients is mainly caused by ZO-1 translocation in the crypt and bacterial overgrowth in the small intestine. In general, NASH and NAFLD are highly associated with impaired TJ integrity [23].

### 5.5. Chronic Heart Disease

Heart failure is the inability of the heart to pump enough blood to the body, so it cannot deliver the necessary oxygen and nutrients to the rest of the body. Chronic heart failure is the most common and develops gradually over months or years [52]. Patients with chronic heart failure showed a 35% increase in small bowel permeability with the lactulose/mannitol test and a 210% increase in large bowel permeability with the sucralose test. These increases in permeability were associated with disease severity, venous blood congestion, and serum C-reactive protein (CRP). In addition, high levels of endotoxins and inflammatory cytokines such as TNF and STNF-R1 were found. A study of the gut microbiota in such patients showed that they had massive amounts of pathogenic bacteria such as Campylobacter, Salmonella, and Candida compared to healthy subjects. All this indicates that an alteration of the barrier function in patients with CHF can induce translocations of bacteria and trigger the generation of cytokines, thus contributing to a deterioration of cardiac function [53].

### 5.6. Celiac Disease

Celiac disease is a disease of autoimmune origin with a hereditary component caused by the ingestion of cereals that contain gluten. After the ingestion of gluten in celiac patients, gliadin, a glycoprotein present in cereals, crosses the epithelium and reaches the macrophages of the intestinal submucosa, where a response is initiated by proinflammatory molecules that recognize the protein as a cytotoxic agent and cause intestinal inflammation and increased permeability [54]. This response can cause structural alterations in the TJs. It has been shown that the increase in intestinal permeability is due to an increase in the protein zonulin, which modulates tight junctions and paracellular permeability. Although gluten can trigger the release of zonulin in both healthy individuals and celiacs, the amount of zonulin produced is much higher in the latter. Celiac disease increases intestinal permeability and, consequently, induces a reorganization of the cytoskeleton through PKC and the disruption of the integrity of the tight junctions [55].

### 5.7. Type 1 Diabetes Mellitus

Diabetes mellitus is a chronic disease caused by an inability of the body to synthesize insulin or by the appearance of insulin resistance. Type 1 diabetes is characterized by an autoimmune response against the host’s pancreatic β cells, leading to insufficient insulin production [56]. Certain studies indicate that there could be a relationship between intestinal barrier dysfunction and type 1 diabetes mellitus. Firstly, studies in humans with type 1 diabetes mellitus show impaired intestinal barrier function, even before the onset of the disease, and increased intestinal permeability due to the production of zonulin. On the other hand, recent studies indicate that microbial translocation contributes to the development of type 1 diabetes. Together, the results suggest an essential role of intestinal permeability in the progression of type 1 diabetes [57].

## 6. Factors That Influence Intestinal Permeability

### 6.1. Dysbiosis

The dynamic interactions between the gut microbiota and the immune system are significant for maintaining intestinal homeostasis and inhibiting inflammation, as well as for understanding the importance of dysbiosis [58]. They have been attributed several functions to the microbiota related to the regulation of intestinal permeability, including the control of the proliferation of pathogenic bacteria, the stimulation of the immune system, the production of short-chain fatty acids, which modulate the host immune system and serve as a carbon source for colonocytes, and fermentation of amino acids and glucosaccharides [59]. The imbalance in intestinal microbiota alters the tight intercellular junctions (TJ) that allow access to pathogens and toxins (bacterial lipopolysaccharides, LPS). Additionally, it induces stimulation of mucosa-associated lymphatic tissue (MALT) with the activation of the inflammatory cascade (leukocytes, cytokines, and TNF-α), the establishment of a chronic inflammation process and, consequently, massive tissue damage [60].

Among the thousands of bacterial species identified in the healthy human intestine, 90% belong to *Proteobacteria*, *Firmicutes*, *Actinobacteria*, and *Bacteroidetes*. A dysbiotic microbiota can be produced by the increase in pathobionts such as *Proteobacteria phylum*, *Escherichia*, *Vibrio*, *Yersinia*, *Helicobacter*, and *Salmonella*, and the decrease in commensals such as Clostridium group IV and XIVa, *Bacteroides*, *Bifidobacterium* or *Faecalibacterium prausnitzii* [61]. The composition of the microbiota changes continuously throughout life, and many factors influence its composition. Thus, it mainly varies according to factors such as diet, age, genes, drugs ingested, and environmental, physical, and psychological stress [49].

### 6.2. Infections

Infections can also play a role in disrupting the intestinal barrier. For example, *Helicobacter pylori* infect the human stomach. This bacterium is known to increase intestinal permeability due to the redistribution of the ZO-1 protein from the TJ [62]. Additionally, it has been found that bacteriophages, which were not generally considered mammalian pathogens, may have some impact on the leaky gut [57]. The pathological effect of these bacteriophages manifested as an increase in intestinal permeability and the translocation of bacterial components and products. Bliss translocation is considered among the main triggers of various polyetiological diseases associated with chronic inflammation and leaky gut. However, due to the possible association of bacteriophages with leaky gut, it may be caused by phages found in the intestinal microenvironment to which humans are continuously exposed. The infection of the microbiota by bacteriophages represents a new group of viral diseases in mammals. Even so, further studies should be carried out to confirm the effect of bacteriophages on the intestinal microbiota and evaluate its implications in the different human pathologies [63].

### 6.3. Antibiotics and Drugs

The gut microbiota can also be affected by antibiotics or other drugs. A study on the effects of antibiotics with different modes of action on the composition of the human microbiota showed that antibiotic treatment could increase or decrease certain species of the intestinal microbiota [64]. Macrolides are among the most widely used antibiotics in children and adults. It has been shown that consumption of antibiotics, for a prolonged time, in children led to an alteration in the intestinal microbiota, which decreased *Actinobacteria* and increased *Bacteroides* and *Proteobacteria*. On the other hand, clarithromycin, the first antibiotic used to eradicate Helicobacter *pylori*, showed a decrease in *actinobacteria* and *firmicutes*, with an increase in *Bacteroides* and *Proteobacteria* after *H*. *pylori* eradication. Some studies showed that vancomycin decreased fecal microbiota diversity due to reduced *Firmicutes* and increased *Proteobacteria*. Ciprofloxacin was found to reduce *Firmicutes* and *Actinobacteria* (specifically *Bifidobacterium*) and increased *Bacteroides*, while clindamycin decreased *Lactobacillus* and *Bifidobacteriaceae* [65]. In addition, other drugs such as nonsteroidal anti-inflammatory drugs (NSAIDs), aspirin, or paracetamol damage the gastric and intestinal mucosa and are associated with gastrointestinal complications. Patients who are long-term users of these drugs may show a decrease in absorption capacity and a possible increase in bowel permeability [66].

### 6.4. Alcohol

Animal studies have shown that alcohol could cause increased intestinal permeability, depending on the dose and time of administration of alcohol, and decreases in the hydrophobicity of the mucosal surface (a physiological marker of mucosal barrier function) associated with increased levels of free fatty acids in the intestinal lumen. These results suggest that alcohol can cause loss of intestinal barrier function by extracting and dissolving intestinal mucosal lipids with a resulting decrease in the hydrophobicity of the mucosal surface, which is a critical component of intestinal barrier function [67]. Human studies support the inhibition of beneficial bacteria and the dysbiosis produced after high consumption of alcohol. Alcohol consumption has been shown to alter the mucosa-associated microbiota composition in the sigmoid colon biopsies [68].

### 6.5. Stress

On certain occasions, stress can affect the development of the intestinal barrier and be associated with increased gut permeability [69]. An example of this type of stress is burns and alcohol consumption, which we have referred to in the previous section. Burn injury, mediated by myosin light-chain (MLC) kinase activity, results in an increase in gut permeability. In addition, MLC phosphorylation or activation of other kinases triggers the opening of TJ proteins (including ZO-1 and claudin-1), which can be reversed by adding an inhibitor of MLC phosphorylation [57]. The effects of stress on intestinal permeability are not simple and possibly involve the brain, apart from the gut. Corticotropin-releasing factor (CRF) and its receptors (CRFR1 and CRFR2) play a key role in permeability dysfunction in the stress-induced gut. In response to an acute stressor, paracellular permeability is associated with visceral hypersensitivity. In turn, stress in early life enhances plasma corticosterone in rat pups. It is associated with increased intestinal permeability and bacterial translocation to the liver and spleen, predominating the effect in the colon [70]. Human studies also confirm that acute stress can affect gut permeability [70]. A stressor such as public speaking produces an increase in intestinal permeability with an increase in cortisol levels. Another stress factor, pain due to cold, produces increased permeability to albumin, although it is only found in women [70]. The stress factor is also relevant in the prenatal period since babies of mothers with high stress reported high concentrations of salivary cortisol during pregnancy and had a significantly higher relative abundance of *Proteobacteria* and a relative abundance of minor lactic acid bacteria (*Lactobacillus*, *Lactococcus*, *Aerococcus*, and *Bifidobacteria*). Likewise, babies with an altered composition of the microbiota showed a higher incidence of gastrointestinal symptoms and allergic reactions in children, highlighting the functional consequences of aberrant colonization patterns in the early years of life [70].

### 6.6. Diet

The diet strongly influences the microbial composition and functions of the gut. Diet has a dominant role in the configuration of the intestinal microbiota since dietary components can significantly alter gastrointestinal functions, compromising the intestinal barrier’s integrity [71]. The consumption of carbohydrates and lipids can affect intestinal permeability, leading to bowel syndrome permeable [7]. Additionally, the different eating habits in different countries are reflected in the incidence rates of intestinal diseases. The intestinal hyperpermeability cases tend to be located in countries with a Western culture, where a diet rich in fats and refined carbohydrates predominates [72]. The carbohydrates and liquids induce cellular inflammation through intestinal dysbiosis and affect both the metabolism of the gastrointestinal tract of the host and immune homeostasis [73]. Long-chain fatty acids are critical components of living cells and have been shown to influence intestinal permeability due to the alteration of TJs and the acetylation of histones. Several studies showed that fructose, glucose, and sucrose are implicated in increased intestinal permeability and dysfunction of TJs [7].

**Figure 5:**
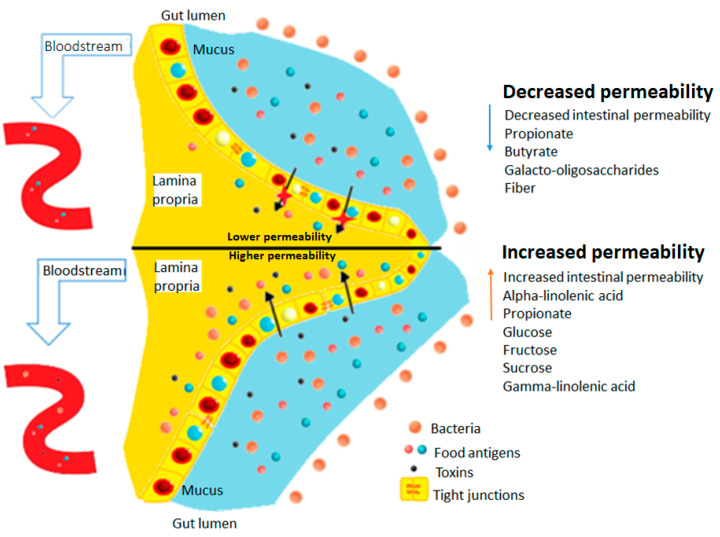
Effect of various components of the diet on the permeability of the intestinal epithelium. The components that decrease intestinal permeability appear on the upper part of the figure and those that increase it appear on the lower part of the figure.

On the other hand, some carbohydrates, such as galactooligosaccharides, can support the growth of beneficial bacteria, and dietary fiber has been shown to positively affect intestinal permeability [7]. The dietary fiber is digested by enzymes and microorganisms fermenting short-chain fatty acids such as butyrate and propionate, which are key factors in the protection of the intestine [73]. Additionally, food additives have been related to permeable bowel syndrome. A recent review describes the ability of additives to increase intestinal permeability by interfering with TJs, promoting the passage of antigens immunogenic to the organism [74]. A potentially beneficial diet would focus on avoiding products such as fruits abundant in fructose, food additives, and oils that contain ALA and GLA. On the other hand, patients with LGS should consume a greater amount of dietary fiber and less sugars and fats (Figure 5) [7].

## 7. Ingredients That Help Treat Leaky Gut

### 7.1. FODMAP

FODMAP is a collective term that consists of fermentable oligosaccharides, disaccharides, monosaccharides, and polyols [75]. Intestinal permeability has also been associated with the pathogenesis of irritable bowel syndrome (IBS), which is treated with FODMAP. IBS patients showed lower protein zonula occludens (ZO)-1 and occludin in intestinal tissue. They showed an increase in the production of cytokines in peripheral blood mononuclear cells (PBMCs), and these patients (most notably those with diarrhea) showed basal levels of elevated serum TNF-α, IL-1β, and IL-6 [4]. The first study demonstrating the function of a low-FODMAP diet in handling gastrointestinal problems was clinical trials with IBS and fructose malabsorption on a low-fructose/fructan diet [75]. Fructans and galactooligosaccharides have well-documented prebiotic actions [76].

Nevertheless, fructose over glucose, lactose, sorbitol, mannitol, fructans (fructooligosaccharides, inulin), and galactooligosaccharides have shown osmotic action, increasing small bowel and lumen water content. Slow or no absorption of FODMAPs results in an increase in osmotic action, resulting in increased luminal water content and subsequent distention of the small intestine, leading to IBS symptom induction [76]. Malabsorption of FODMAPs results in their delivery to the large intestine, allowing exposure to the microbiota and subsequent fermentation, resulting in gas production and luminal distention of the large intestine, leading to IBS symptom induction. The low-FODMAP diet has been recommended to provide irritable bowel syndrome patients with a treatment approach that can effectively relieve most symptoms in most patients and is now supported by high-quality evidence, including randomized controlled trials [77].

FODMAP consumption affects multiple gastrointestinal effects. Some of them may be beneficial, such as increased fecal bolus volume, improved calcium absorption, and increased production of short-chain fatty acids. Selective stimulation of some components of the microbiota, such as bifidobacterium, is also described, as well as a positive effect on the growth and function of the intestinal microbiota [77].

### 7.2. Probiotics

**Figure 6:**
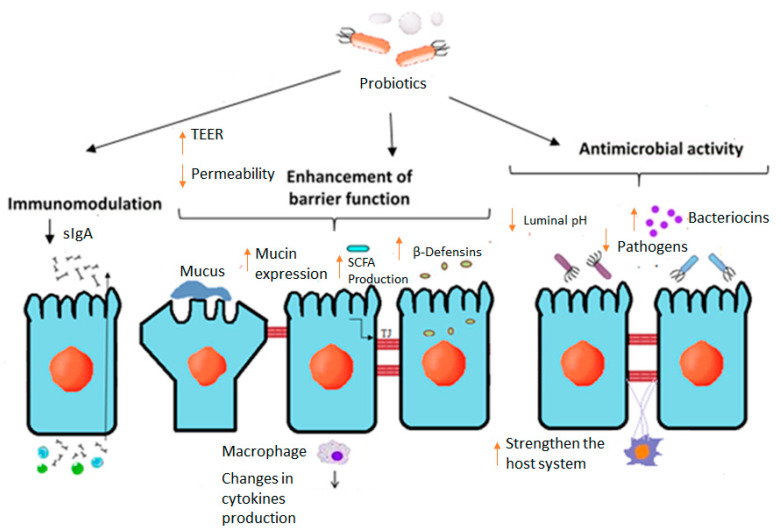
Effects of probiotic bacteria on intestinal epithelial barrier function. Figure adapted from Ohland and MacNaughton (2010) with modifications.

Probiotics are viable microorganisms with physiological or beneficial therapeutics. Probiotics are found both in foods and as supplements, with the most common foods being yogurt and kefir [78]. Among the main effects of the administration of probiotics are the maintenance of homeostasis and intestinal integrity, regulation of intestinal transit, the production of short-chain fatty acids and vitamins, and providing enzyme digestion activities for the degradation of undigested fibers and the neutralization of xenobiotics. Probiotics could also help modulate intestinal permeability affecting the mucus, epithelium and microbiota (Figure 6).

Probiotics can exhibit anti-inflammatory properties against TNF-α or IL-6 [79]. They can also strengthen the mucosal barrier [80] and reduce intestinal permeability, upregulating TJS proteins [81]. The advantageous effect of probiotics in pouchitis is associated with the homeostasis of the mucosal barrier [82]. Another possible mechanism of action is the addition of butyrate-producing species [83]. These factors combine to result in greater integrity of the intestine, making probiotics a fantastic therapy for reducing leaky gut [84]. Table 1 illustrates the major intestinal epithelial homeostasis and health benefits of probiotics.

Various species of *Lactobacillus* exert effects on the expression and secretion of mucins. Intestinal mucins are the mucus’s main protein component that covers the gastrointestinal tract’s epithelium. These glycosylated macromolecules (up to 80% *w*/*w*) are synthesized by goblet cells or goblet cells. They are located in the cell membrane or secreted into the intestinal lumen to form the mucosal layer. Of the 18 mucin-like glycoproteins expressed by humans, the mucin MUC2 is the predominant glycoprotein found in the mucus of the small and large intestines. The NH2 and COOH termini are not glycosylated to the same extent but are rich in cysteine residues that form intra- and intermolecular disulfide bonds. These glycan groups confer proteolytic resistance to the mucins, while the disulfide bonds form a matrix of glycoproteins that is the backbone of the mucosa. This layer protects the epithelium against antigens and potentially harmful molecules and acts as a lubricant for intestinal motility. The mucus is the first barrier intestinal bacteria encounter, and pathogens must penetrate it to reach epithelial cells during infection. Microorganisms have developed various mechanisms to degrade mucus, such as mucin disulfide bond reduction, proteolytic activity, and glucosidase valuable activity to invade or absorb nutrients derived from mucus. On the other hand, the colon’s mucosal layer is thinner in areas of inflammation, allowing greater adherence and bacterial infiltration.

In vitro studies show that various species of *Lactobacillus*, such as *L*. *plantarum* 299v, *L*. *rhamnosus* GG, and *L*. *acidophilus* DDS-1, may increase mucin expression and secretion by goblet cells as a mechanism to enhance barrier function and pathogen exclusion by limiting bacterial movement through of the mucosal layer [1,115,116].

Another positive effect of some probiotic microorganisms on the epithelium is the increase in the expression and secretion of defensins. α-defensins (HD-5 and HD-6), expressed mainly by Paneth cells in the small intestine, and β-defensins (hBD1 to hBD-4), expressed by epithelial cells throughout the intestine, possess antimicrobial activity against a wide range of variety of bacteria, fungi, and some viruses and are constitutively expressed to prevent pathogens from reaching the epithelium. For its part, the decrease in the production of defensins has been associated with the development of inflammatory bowel disease and greater susceptibility to bacterial infections [1]. Various species of the Lactobacillus genus and commercial probiotic preparations have shown in in vitro studies with Caco-2 cells and in vivo studies with humans that they can regulate the expression and secretion of β-defensin hBD-2. Increased defensin expression and mucus secretion by epithelial cells may prevent the proliferation of commensals and pathogens, thus also contributing to barrier integrity [117,118]. Patients who received the commercial preparations twice daily for three weeks showed a significant increase in fecal levels of hBD-2 protein, while individuals treated with a placebo showed no change [118]. These levels were maintained for 9 weeks after cessation of probiotic treatment, although to a lesser extent [118].

Probiotics can increase the levels of immunoglobulin A (IgA)-producing cells in the lamina propria and promote the secretion of secretory IgA (sIgA) in the luminal layer of the mucosa. These antibodies limit epithelial colonization by binding to bacteria and their antigens, contributing to intestinal homeostasis. Some studies show that certain microorganisms can increase the levels of total and pathogen-specific IgAs after infection, without increasing probiotic-specific IgAs. Galdeano and Perdigón (2006) [119] showed that the administration of *L. casei* to mice significantly increased the number of IgA- and IL-6-producing cells, which can stimulate class switching to IgA in B cells within the lamina propria of the mouse. In addition, they did not find specific antibodies against *L. casei,* which indicates the lack of response of the intestinal immune system to this beneficial bacterium [119].

Different tests have experimented with the effectiveness of various species of probiotics in intestinal permeability, including *Lactobacillus rhamnosus* GG, *Lactobacillus acidophilus*, *Lactobacillus Plantarum*, *Bifidobacterium infantis*, *Bifidobacterium animalis lactis* BB-12, and *Escherichia coli Nissle* 1917 [120] (Table 1). Table 1 details the role of probiotics in major epithelial intestinal modulations and possible mechanisms of actions in the intestinal barrier that eventually can potentially provide health benefits for leaky gut syndrome-related diseases. Comprehending the mechanism of action of the dietary nutrients may assist in delivering knowledge about the potential of the ingredient and how it affects intestinal barrier dysfunctions. Nevertheless, the probiotic application is still limited, and their mechanisms of action are not fully understood.

### 7.3. Vitamins

For the most part, vitamins A and D play critical functions in regulating gastrointestinal homeostasis [121]. In clinical trials, these vitamins impacted the components of the mucosal barrier, including epithelial integrity, immune system, and gut microbiota. It is suggested that vitamins A and D’s effects on gut microbiota composition are indirect [122]. In animal studies, these vitamins reduce microbial diversity and increase the Proteobacteria phylum [123], which are potentially pathogenic in patients with inflammatory bowel disease (IBD) [124]. In human studies, vitamin A-sufficient children have more diverse microbial communities when compared to vitamin A-deficient children [125]. In the intestinal epithelium, in vitro studies show that vitamin A and vitamin D improve the tight junctions (ZO-1, occludin, and several claudins) [126,127]. Vitamins A and D are necessary for the integrity of the epithelium and gut microbiota and modulate immune responses at different levels. Both vitamins can inhibit T-cell IFN-γ production [128] and inhibit Th17 cells in vitro. In vitro models, these vitamins can generate IL-10 production and FOXP3 protein, which is implicated in immune system reactions [129]. Retinoic acid can stimulate the production of antibacterial peptides such as Reg3β and Reg3γ in enterocytes [130]. These and other results indicate that the theoretically advantageous effects of vitamins A and D may be because of the regulation of different elements of the mucosal barrier. Nevertheless, only a few studies or clinical trials are available in the literature, and more research is encouraged.

### 7.4. Fibers and Short-Chain Fatty Acids

Among carbohydrates, dietary fibers (DF) are appropriate for anti-inflammation properties and intestinal barrier regulation. Accordingly, the microbiota ferments DF and produces short-chain fatty acids (SCFAs) such as butyrate, propionate, and acetate. In particular, the beneficial species of *Bifidobacterium bacteria* and *Lactobacilli* are related to the production of SCFA and the immunostimulation and inhibitory effects on the growth of harmful bacteria [131]. The SCFA constitutes the primary energy source for the colonocyte’s epithelial cells. Butyrate is the most critical substrate the colonocyte prefers, providing between 60 and 70% of the energy requirements [132]. SCFAs are critical in metabolism, immunity, and intestinal barrier functions. For instance, butyrate can improve paracellular permeability by modulating hypoxia-inducible factor-1 and epithelial tight junction CLDN1 [133]. In addition, butyrate regulates the central element of the colonic mucus layer, goblet-cell-specific mucin MUC2 expression in human goblet cell-like LS174T cells [134]. Regarding its anti-inflammatory properties, SCFAs modulate the chemotaxis of immune cells and release reactive oxygen species (ROS) and cytokines. SCFAs could have a binding regulatory effect on inflammatory diseases by controlling the migration of immune cells towards the site of inflammation and modulating their activity, allowing the rapid elimination of pathogens by activating ROS. The above binding process can contribute to the reduction in damage to the host, which could allow not only its survival but also the production of SCFA by intestinal bacteria [135].

The deficiency of SCFAS and dietary fibers can compromise epithelial and mucus barrier functions by increasing gut permeability. In mice, the deficiency in consumption of dietary fibers and SCFAS production can harm intestinal barrier integrity by inhibiting *Akkermansia muciniphila* [136]. This bacterium uses mucus glycans as a nutrient source without dietary fibers. This alteration can cause damage to the colonic mucus barrier and result in the development of colitis caused by the enteric pathogen *Citrobacter rodentium* [136]. Yamada et al. (2015) [137] evaluated changes in SCFAs in 140 patients diagnosed with severe systemic inflammatory response syndrome (SIRS) and compared them with healthy volunteers; they found a significant decrease in the concentration of butyric, propionic, and acetic acids and a significant increase in pH; both the total concentration of organic acids and SCFA were significantly lower in patients with gastrointestinal dysmotility [137]. In this study, regarding the healthy volunteers, the authors reported in inflammatory response syndrome patients a significantly lower count of obligate anaerobic bacteria (*Bacteroidaceae*, *Bifidobacterium*, and *Enterobacteriaceae*) and a significantly higher count of facultative anaerobic microorganisms (*Enterococcus*, *Staphylococcus*, *Pseudomonas*, and *Candida*). The mechanisms by which SCFA decrease in critically ill patients are not precise. Although more studies are needed, the decrease in obligate anaerobic bacteria may affect their concentration in the long term [138]. The fermentation substrates necessary for SCFA production in these patients may be decreased [139]. An increase in intestinal permeability and a decrease in pH has been reported in critically ill patients [140].

### 7.5. Glutamine

Dietary glutamine is not subject to significant acid hydrolysis in the stomach and upper duodenum and is, therefore, effectively available in the small intestine for absorption and metabolic utilization. In accompaniment to its essential function in absorption, secretion, and digestion, amino acids are critical to nourishing gut health as a barrier to the permeability of pathogens, allergens, and toxins into the epithelium. Glutamine is considered a crucial amino acid capable of regulating the expression of tight junction proteins, allowing the membrane of intestinal cells to remain impermeable. Preclinical studies have indicated that adding glutamine improves fibrosis and intestinal inflammation [141,142]. In T and B-lymphocytes and epithelial cells, glutamine improves anti-inflammatory IL-10 levels and decreases the production of pro-inflammatory IL-6 and IL-8 [143]. Since IL-10 is vital in sustaining intestinal mucosal homeostasis, this amino acid is considered a regulator of the innate and adaptive immune response system [144]. Glutamine with probiotics can have beneficial effects when treating intestinal permeability in patients with severe disorders. This combination could generate a synergistic effect by increasing the amount of *Lactobacillus*, slowing down the growth of Gram-negative bacteria, improving the structure of the intestinal flora, and restoring damage to the colonic barrier. In addition, it can effectively reduce the intestinal mucosa’s permeability and the intestinal endotoxin level, restoring the mechanical damage of the intestinal barrier and thus reducing intestinal bacteria’s translocation [145]. However, research evaluating the impact of glutamine on the intestinal barrier in preclinical models is restricted. It is recommended to demonstrate that nutritional intervention can impact the clinic by approaching treatment on time and avoiding surgical complications. In addition, it is essential to implement a guide on the perioperative nutritional management (encompasses preoperative, intraoperative, and postoperative care) of patients with gastrointestinal disorders, detailing the importance of using glutamine, dosage, and probiotics strains. A Randomized Clinical Trial (RCT) with a larger sample size is suggested to confirm the positive benefits of using probiotics and glutamine. Even though multiple in vitro, in vivo, and clinical studies reveal that glutamine has an advantageous function in the maintenance of the mucosal barrier, larger randomized trials are required to evaluate the advantageous effects of glutamine.

### 7.6. Arginine

Unlike glutamine, just a few studies indicate the protective effects of arginine on intestinal epithelium integrity. In an intestinal obstruction model, arginine decreased intestinal paracellular permeability (400 Da molecule) and *E. coli* translocation [146]. Similarly, l-arginine (4 mM) moderately inverted the increase in paracellular permeability of Lucifer Yellow (457.25 Da) and the reduction in TEER (transepithelial electrical resistance) generated by heat stress [147]. Protective effects of L-arginine have been reported on the intestinal epithelial barrier under heat-stress conditions in rats and the IEC-6 cell line [148]. In IPEC-J2 cells, arginine improved hypoxia-induced paracellular inulin permeability, decreased TEER, and moderation ZO-1 [149]. In the small intestine of nonalcoholic steatohepatitis rodents, arginine regulates occludin and ZO-1 and promotes plasma levels of bacterial endotoxins [150]. Arginine enhances the intestinal barrier function of birds fed a reduced protein diet under stress conditions [151]. Similarly, arginine demonstrated favorable effects in overall growth, intestinal integrity, and morphology in broilers subjected or not to the *Eimeria* challenge. In the intestinal skin of mice, arginine increased the *Bacteroidetes* population and the production of mucin-2, mucin -4, TNF-α, IL-1β, IFN-γ, paneth antimicrobials, and secretory immunoglobulin A [152]. Well-designed clinical trials must be addressed to confirm the potential observed in preclinical trials.

### 7.7. Polyphenols

To date, the mechanisms of action regarding polyphenols with intestinal permeability have not been well understood. Nevertheless, polyphenols are involved in a direct/indirect matter with intestinal permeability by NF-κB inactivation, a pathway identified as among the most significant arbitrators of cytokines, interleukins, and inflammation. Furthermore, the NF-κB activation is related to impairing the epithelial barrier function by TJ disassembly. Luescher et al. (2017) [153] have reported that polyphenols inactive NF-κB by containing degraded proteasome of IκB and interfering with IκB kinase phosphorylation [153]. Another essential aspect potentially engaged in improving intestinal epithelium functions is the inhibition of several protein kinases, including activated protein kinase, phosphoinositide-3-kinase, tyrosine kinase, mitogen-activated protein kinase, myosin light-chain kinase, protein kinase C and adenosine monophosphate [154]. Some epigallocatechin 3-gallate, curcumin, and quercetin have been reported to decrease intestinal permeability by inhibiting protein kinase C and myosin light-chain kinase involved in the phosphorylation of inflammatory proteins [155,156,157].

Initially, the beneficial effects of polyphenols were attributed to their ability to eliminate ROS, that is, antioxidants. There is increasing evidence that its benefits are strongly related to the ability to interfere with redox signaling pathways [32]. it is considered that oxidative stress could be involved in the etiology of intestinal permeability; polyphenols, due to their properties, are proposed for treating the disease. Research suggests that the diet’s consumption of polyphenols contributes to restoring redox homeostasis and increasing the activity of antioxidant enzymes (SOD, CAT, GPx, and GR). The expression of these enzymes is regulated by a nuclear erythroid-derived factor 2 (Nrf2). Nrf2 is activated at the cellular level by ROS and translocates to the nucleus, where it regulates the transcriptions of various genes encoding the aforementioned antioxidant enzymes. The antioxidant activity of polyphenols is associated with the ability to activate Nrf2 and, therefore, regulate antioxidant enzymes [158].

A critical aspect that must be highlighted in this broad group of metabolites is their bioavailability since it is essential to evaluate their biological properties. It is reported that, after ingesting polyphenols, only a percentage between 1 and 10% of the total is detected in urine and plasma samples. Although this group of compounds generally has low oral bioavailability, some subgroups of the highest classification differ in this parameter. For example, bioavailability is particularly low for flavones, while it is higher for flavanones and soy isoflavones. Thus, consuming 10–100 mg of simple polyphenol results in a plasma concentration rarely exceeding 1 μM. However, the low bioavailability of polyphenols may not be a problem in intestinal diseases since several studies suggest that the highest levels of polyphenols in the human body are concentrated in the intestine [32,159].

Polyphenols have been shown to enhance tight junction integrity, increase mucus secretion, and decrease intestinal barrier permeability, thereby generally improving the intestinal defense mechanism [156,160]. In addition to, the involvement of polyphenols in multiple inflammatory signaling pathways, they also exert beneficial effects by acting on the intestinal epithelium. Table 2 illustrates the polyphenols’ primary intestinal epithelial homeostasis and regulations and health benefits. Table 2 details the role of polyphenols in possible mechanisms of actions that can potentially provide health benefits for intestinal permeability-related illnesses. Recent studies indicate that a polyphenol-rich diet lowers the risk of intestinal barrier dysfunctions. Polyphenols such as quercetin, epigallocatechin gallate, catechin, epicatechin, berberine, resveratrol, and curcumin have been studied intensely to provide health benefits in leaky gut-related diseases.

### 7.8. Medical Herbs

The World Health Organization (WHO) estimated that more than 80% of the world population uses traditional medicine to meet their needs in primary care, using plant extracts or their active ingredients [47]. Herbal medicine can be applied and administered as tea infusions, tea decoctions, alcohol extracts, nonalcohol extractions, percolations (tinctures), elixirs/cordials (pleasant-tasting extractions), capsules, spagyrics, salves, hydrosols, and essential oils [197]. Medical plants have phytochemicals such as organic acids, flavonoids, iridoid glycosides, saponins, chlorogenic acid, secoiridoids, berberine, sesquiterpene, and sesquiterpenoid [197]. These phytochemicals have proven to treat diseases such as obesity, nonalcoholic steatohepatitis, ulcerative colitis, Crohn’s disease, food allergies, inflammatory bowel disease, and irritable bowel syndrome [198]. As noted before, a leaky gut is usually related to diseases such as dysbiosis, immune system imbalance, IBS, and nutritional deficiencies. Herbs are useful for medical therapy and practical nutrition to help leaky gut-associated illnesses [199]. Medical herbs are often used to treat leaky gut-associated autoimmune disorders such as ulcerative colitis, systemic lupus erythematosus, and rheumatoid arthritis [200]; nevertheless, the possible mechanism of action and its effectiveness remain ambiguous. Given the influence of intestinal permeability on numerous illnesses, the integrity of the gut epithelial functions is essential for maintaining intestinal homeostasis. The mechanism of actions in medical plants is broad, including regulating intestinal microbiota and permeability, upregulating both mRNA and protein expressions of claudin-1, lowering extracellular signal-regulated kinase activation in hepatocytes, suppressing MLCK-MLC phosphorylation signaling pathway, and protecting on IECs against LPS-insult [198]. Recent studies suggest that herbs alleviate leaky gut-related diseases in animal studies and clinical trials [198]. Medical herbs can deliver a potential therapeutic approach for gastrointestinal disorders. Table 3 illustrates the most predominant studies concerning medical herbs in leaky gut-related illnesses and its possible mechanisms of actions, modulation and regulation concerning the leaky gut syndrome. Current studies reveal that medical herbs maintain satisfactory gut health, which is related to intestinal disorders.

### 7.9. Mushrooms

The use of mushrooms as functional ingredients has grown in the past decade. Mushrooms are a great source of bioactive compounds such as ergosterol, vitamin D, phenolic compounds, terpenes, and terpenoids. In addition, mushrooms are deemed a viable source of prebiotics as they contain different polysaccharides, such as chitin, chitosan, hemicellulose, xylans, mannans, galactans, and α- and β-glucans [228]. For health relevance, mushrooms are considered to pose a critical function in immunoregulating antitumor activities, atherosclerosis, and pneumococcal pneumonia. In leaky gut-related diseases, mushrooms have been demonstrated to potentially treat pancreatitis, nonalcoholic fatty liver disease, colitis, obesity, and diabetes [229]. Mushrooms were found to modulate gut microbiota by stimulating the production of catecholamines, their metabolites, and the inflammatory response. As mentioned, mushroom polysaccharides can also affect SCFAs production mainly butyrate, propionate, and acetate [230]. Many studies have reported within the lower intestinal tract that mushroom polysaccharides contributed to the proliferation of *Bacteroidetes*, which is liable for most acetate and propionate production [231]. The SCFAs can interact with many receptors to enhance immune-signaling and anti-inflammatory activities. SCFAs can function as signaling molecules with G-protein-associated receptors such as free fatty acid receptor 3 and free fatty acid receptor 2 to discharge GLP-1 and GLP-2 that regulates the tight junctions [232]. Table 4 illustrates the most predominant studies concerning mushrooms in intestinal epithelial homeostasis, health benefits, and gut microbiota regulation. Table 4 is aimed at collecting the health-promoting advantages of edible mushrooms via the gut microbiota. Recent studies showed that mushrooms act as prebiotics to promote a balanced and healthy gut microbiota, granting health benefits to leaky gut-related diseases.

## 8. Other Foods That Can Potentially Help Treat Leaky Gut

When examining foods in leaky gut-related diseases, the composition of the gut microbiome and gut-derived metabolites produced by functions are intensely interesting. In addition, ingesting functional food such as yogurt can influence the gut microflora, which impacts intestinal epithelial homeostasis [263,264,265]. In food matrixes, the influence of polyphenols, fat, proteins, carbohydrates, and pre/probiotics on microbiota has also been investigated [199,266]. For this reason, we will not repeat the studies performed on macromolecular food components. Rather, we will illustrate the current studies of the food systems in intestinal homeostasis to show the great impact of dietary whole-food consumption on maintaining intestinal integrity.

Recent studies have shown that small-molecular food phytochemicals such as glycyrrhetinic acid and polydatin can protect intestinal epithelium [208,267]. Therefore, there is an urgent need to summarize the detailed effect of foods on intestinal aliments at different stages. Table 5 focuses on the in vivo effects of foods and their phytochemicals and illustrates their effects on every step of the intestinal pathological progression, from intestinal epithelial atrophy to colorectal cancers, to demonstrate their potential as dietary supplements regarding intestinal health. Table 5 details the role of foods and ingredients in possible mechanisms of actions in the intestinal barrier that eventually can potentially provide health benefits for leaky gut syndrome-related diseases. Table 5 describes functional foods and ingredients that promote intestinal epithelial regeneration by exercising intestinal-functions effects, interfering with intestinal inflammation, and improving intestinal flora. Nevertheless, most of these studies are based on preliminary in vitro studies, such as in vitro anti-inflammatory and cytotoxic studies. The results cannot reveal the real potential of foods in modulating human bio-functions. In addition, concerning intestinal diseases, studies on functional foods mainly focus on macromolecules such as prebiotics and polysaccharides. In contrast, the effects of small-molecular constituents of foods are less investigated and should be addressed in future studies.

## 9. Conclusions

The intestinal microbiota is essential to maintain the intestinal epithelium’s integrity and homeostasis. A qualitative and quantitative imbalance in the composition of the intestinal microbiota or dysbiosis contributes to intestinal barrier dysfunction and leaky gut syndrome. Certain infections, an unhealthy diet, stress, excessive use of antibiotics and other drugs, and alcohol can influence increased intestinal permeability and cause leaky gut syndrome. The intestinal hyperpermeability produced in leaky gut syndrome leads to an alteration of the tight junctions, the entry of toxic agents into the blood, and dysfunction in organs and systems. Leaky gut syndrome therapy should include diet modification avoiding fats, sugars, additives, and ultra-processed foods, and the appropriate supplementation of probiotics/prebiotics, arginine, glutamine, polyphenols, vitamins, fibers, medical herbs, edible mushrooms, and FODMAPs. Several studies have shown that these ingredients influence the modulation of intestinal immunity, regulation of the intestinal epithelial barrier, amelioration of mucosal abnormalities, and growth of epithelial cells. This review delivers recent insights into the critical functions of the dietary ingredients proposed to maintain intestinal barrier functions. Nevertheless, most of the studies are based on animal models, and more well-designed clinical trials are required to address the potential of these ingredients when regulating intestinal barrier dysfunctions.

## Figures and Tables

**Table 1:** Major probiotics that help leaky gut.

Probiotic	Major Intestinal Epithelial Regulation	Major Potential Health Benefits
*Lactobacillus rhamnosus GG*	Increased claudin-3 in timed-pregnant mice [81]. Preserves zonula occludens-1 and myosin light-chain kinase in vivo [85]. Inhibits LPS or TNF-α in CaCo2 cells [86]. Downregulation of (TNF)α and (IFN)γ, enhanced transcription of IL-10 in mice [87].	Improves gastrointestinal disorders/irritable bowel syndrome in children [88] and adults with supplementation of a low-FODMAP diet [89]. Improves diarrhea and gastroenteritis in children [90,91]. Improves colitis in mice [87].
*Lactobacillus acidophilus*	Increased occludin in rats [92]. Reduces colonic polyps and systemic inflammation and increases anti-inflammatory regulatory T cells in mice [93].	Improves acute diarrhea in children [94] and chronic diarrhea in adults [95].
*Lactobacillus plantarum*	Increased occludin and ZO-1 in Caco2 cells [96]. Increase in IL-8 mRNA in epithelial cells and downregulation of IL-8 secretion in HT-29 cells [97]. IL10 production modulation in mononuclear cells stimulation and murine model of colitis [98].	Reduction in symptoms IBS in adults [99]. Improves lipid metabolism and lowers weight in rats [100].
*Bifidobacterim infantis*	Preserves apical distribution of claudin-4 and occluding in baby mice (pups) [101]. Induces Foxp3- and IL-10-secreting T cells in human peripheral blood [102]. Reduces inflammation in Cronobacter sakazakii-induced intestinal inflammation in newborn mice and inhibits interleukin (IL)-1β-induced IL-6 induction in immature enterocytes via regulation of TLR-4 signaling [103]. Secretes indole-3-lactic acid that inhibits IL-1β-stimulated IL-8 production in immature enterocytes [104].	With B. longum and B. breve, it improves the antigen uptake in pediatric patients with Crohn’s disease (CD) [105]. Improves mental health in victims with irritable bowel syndrome developed after a major flood disaster [106]. Promotes weight gain in infants with severe acute malnutrition [107].
*E. coli Nissle 1917*	Increased ZO-2 mRNA and protein expression and apical redistribution and increased ZO-1 mRNA and protein expression in Intestinal epithelial cell line T84 and Caco-2 cells [108]. Upregulation of ZO-1, ZO-2 and claudin-14 in T-84 and Caco-2 polarized monolayers [109].	Maintaining remission in ulcerative colitis patients (adults) [110]. Ameliorate DSS-induced colitis in mice [111].
*Bifidobacterium animalis lactis BB-12*	Reduces LPS, D-lactic acid, diamine oxidase, and levels of pro-inflammatory cytokines and increased the expression of aquaporin and tight junction proteins, and the levels of anti-inflammatory cytokines in adult mice [112].	Improves antibiotic-associated diarrhea in adults [113]. Bowel function improvement in adutls [114].

**Table 2:** Major polyphenols that help leaky gut.

Polyphenols	Major Intestinal Epithelial Regulation	Major Potential Health Benefits
Quercetin	Upregulation of occludin, claudin-1, claudin-3, claudin-4, and claudin-7 in Caco2 cells [161]. Upregulation of ZO-1 and occludin in ECV304 cells [162]. Upregulation of claudin-1, claudin-2, claudin-3, claudin-4, claudin-7, and occludin in HT-29/B6 cells [163].	Effective against asthma, in comparison to medications and steroid inhalers in adults [164]. It has a positive impact to prevent and treat the occurrence of diabetes mellitus in adult male albino rats [165]. In hypertensive rats, it enhances antihypertensive properties [166]. It can assist treating obesity, diabetes, and Alzheimer’s disease [167].
Epigallocatechin gallate	Upregulation of IFN-γ in T84 cells [168].	Can assist in treating cardiovascular disease and obesity [169]. Effectiveness in patients with papilloma virus-infected cervical lesions [170]. Treating premalignant lesions in men with high-grade prostate intraepithelial neoplasia [171].
Catechin	Upregulation of ZO-1, occludin, and claudin-4 in T84 cells [160].	In dark tea, exhibits weight-loss properties [172]. Suppresses colon tumorigenesis in mice [173]. Tea catechins assist on influenza infection and the common cold infections [174]. Beneficial effects have been shown with green tea catechins in Alzheimer’s disease and Parkinson’s disease (PD) [175].
Epicatechin	Upregulation of ZO-1, occludin, and claudin-4 in T84 cells [160]. Upregulation of occludin, ZO-1, and claudin-2 in CaCo2 cells [176]. Upregulation of IFN-γ, NOX1/NOX4, FITC, and dextran transport in mice [177].	Can treat obesity-associated comorbidities [177]. In rat hearts, ECG showed cardioprotective effects and could greatly reduce cardiac mortality [178]. In the brain of mice, it reduces Aβ plaques and protect neurons from injuries [179].
Berberine	Upregulation of claudin-1 and claudin-2 in HT29/B6 cells [163]. Upregulation of occludin, claudin-1, ZO-1, and IP in Caco2 cells [180]. Upregulation of claudin-1, claudin-2, claudin-4, and claudin-5 in CaCo2 cells [181].	Potentially, can assist in treating prostate, uterus or endometrium, skin, glioblastoma, bone, oral, liver, gastric, pancreatic and colon cancer [182]. With curcumin, can treat obesity in mice [183]. Ameliorates nonalcoholic steatohepatitis in ApoE-/-mice [184]. Can improve nonalcoholic fatty liver disease in rats [185]. In clinical trials, it improves prediabetes and diabetes mellitus in patients [186].
Resveratrol	Upregulation of claudin-1, claudin-3, claudin-4, claudin-7, occludin, and ZO-1 in IPEC-J2 [187]. Upregulation of ZO-1, and occludin in Caco2 cells [188].	It has a neuroprotective role in the pathology of Alzheimer’s disease [189]. Alleviates ethanol-induced hormonal and metabolic disturbances in the rat [190]. It has anticancer activity potential In vivo and In vitro [189]. Antidiabetic effects in rats [191].
Curcumin	Upregulation of ZO-1, and claudin-1 in CaCo2 cells [192].	Improves weight loss in obese adutls [193]. Improves the memory and cognitive function of Alzheimer’s disease in mice, rats, cats and nonprimates [194]. Improves insulin resistance in skeletal muscle rats [195]. On in vitro model, it can inhibit influenza virus infection and haemagglutination activity [196].

**Table 3:** Major herbs that help leaky gut.

Herbs	Major Intestinal Epithelial Regulation	Major Potential Health Benefits
*Camellia sinensis* (Tea plant)	Improves the intestinal barrier, alleviating dysbacteriosis (reverse 44 of 68 disordered genera), stimulated immunoreactions with significant enhancement of serum TNF-α, IFN-γ, IL-1β, IL-2 and IL-6 of liver and jejunum from mice [201].	The black tea brew has gastro-protection effect in rats [202]. Antiobesity effect in high-fat diet-fed mice and obese diabetic mice [203]. Hypoglycemic effect potential in rats [204].
*Hibiscus sabdariffa* L. (Roselle plant)	Roselle flower extract has been reported to suppress the expression of inflammatory cytokines, including IL-6 and TNF-α in dextran sodium sulfate-induced colitis in mice [205].	Showed a reduction in abdominal fat, serum-free fatty acids, obesity and exhibited improvement in the liver steatosis in obese adults [206]. Showed a positive effect on type II diabetic adults blood pressure [207].
*Glycyrrhiza glabra* (Liquorice plant)	Glycyrrhetinic acid (GA maintains the integrity of the intestinal epithelium via HuR in IEC-6 cells [208]. Studies suggest that licorice may be a mild inhibitor of P-glycoprotein in rat intestinal mucosa [209].	*G. glabra L*. extract can exerted an antiulcergenic effect in an HCl/ethanol-induced ulcer rats [210]. Glycyrrhizin could decrease the content of cholesterol and triglyceride in the plasma of fructose-induced metabolic syndrome-X rats [211]. Hepatoprotective efficacy of nonalcoholic fatty liver disease and hepatitis in randomized controlled trials [212].
*Althaea officinalis* (Marsh mallow plant)	*Althaea officinalis* pretreatment significantly decreased TNF-α and IL-1β and and promotion of mucin, NO, PG-E2, and PG-I2 contents. in stomach glandular rat tissue [213].	Wound healing potential in rabbits [214]. Treats atopic eczema patients [215]. It has antitussive and pain relieving effects and is used in chronic cough, angina and bronchitis [216].
*Zingiber officinale* (Ginger)	Modulates nuclear factor-κB activity and interleukin-1β [217].	It has direct antimicrobial activity and thus can be used in the treatment of bacterial infections [218]. It is used in traditional medicine as therapy against several cardiovascular diseases such as hypertension [219]. It is used for the treatment of various diseases including nausea, gastrointestinal disorders, respiratory disorders, atherosclerosis, migraine, depression, gastric ulcer, and cholesterol; and other benefits of ginger are reducing pain, rheumatoid arthritis, anti-inflammatory, and antioxidant effects [220].
*Mentha piperita* (Peppermint)	The expression levels of CCL2, CXCL1, IL-1β, TGF-β1 and IL-10 genes were upregulated in infected mice model with Staphylococcus aureus and *Pseudomonas aeruginosa* [221].	Peppermint oil assists in treating IBS patients [222]. Peppermint oil and synbiotic lactol (Bacillus coagulans + Fructooligosaccharides) helps treat functional abdominal pain in children [223]. Peppermint extract helps treat the severity of nausea, vomiting and anorexia in patients with breast cancer [224].
*Plantago lanceolate L.* (Ribwort Plantain plant)	Its phenylethanoid acteoside isolate can exhibits antioxidative potential and inhibits 5-hydroxy-6, 8,11,14 eicosatetraenoic acid and leukotriene B. In addition, it inhibits the enzymatic activity of inducible nitric oxide synthase (iNOS) expressed in both macrophages and neutrophils and 5 lipoxygenase [225].	Wound healing effects in mouse model [226]. Its leaf extract has antiseptic ulcer activity in rodents [227].

**Table 4:** Major mushrooms that help leaky gut.

Mushrooms	Major Intestinal Epithelial Regulation	Major Potential Health Benefits	Gut Microbiota Regulation
*Inonotus obliquus* (Chaga mushroom)	Decreased the expression of tumor necrosis factor (TNF)-α, cyclooxygenase (COX)-2, interleukin (IL)-4, interferon (IFN)-γ, signal transducers and activators of transcription (STAT)1 in induced colitis mice [233].	The Fermented Chaga Mushroom (*Inonotus obliquus*) has hypoglycemic and antioxidative effects on streptozotocin-induced diabetic rats [234]. Improves lipid metabolism in mice [235]. Gastroprotective effect in ethanol-induced rats [236].	Its polysaccharide increased the proportion of *Bacteroidetes* and decreased that of *Firmicutes* at the phylum level [237].
*Coriolus versicolor* (Turkey tail)	Inhibits the expression of STAT1 and STAT6 Associated with IFN-γ and IL-4 Expression in ulcerative colitis induced mice [238].	Suppresses inflammatory bowel disease [238]. Protein-bound β-glucan from *coriolus versicolor* has potential for use against obesity in mice [239]. *Coriolus versicolor* aqueous extract ameliorates insulin resistance in Wistar rats [240].	Its polysaccharopeptides increase *Akkermansia muciniphila* population [229].
*Pleurotus eryngii* (King trumpet mushroom)	Decreased the pro-inflammatory cytokines secretion (IL-1β, IL-2, IL-6, IL17A, IFN-γ, and KC) in induced colitis in mice [241].	Suppresses inflammatory bowel disease [241]. Improves postprandial glycaemia, hunger and fullness perception, and enhances ghrelin suppression in people with metabolically unhealthy obesity [242]. Decrease the blood glucose and cholesterol in diabetic rats [243].	Its polysaccharopeptides increase *Anaerostipes* and *Clostridium* population [244].
*Ganoderma lucidum* (Ganoderma)	Suppressed the production of nitric oxide (NO) and prostaglandin E2 (PGE2) in lipopolysaccharide (LPS)-stimulated macrophages and decreased the expression of COX-2, TNF-α, iNOS, IL-1β, IL-6, and IL-10 mRNAs in in a mouse model of colitis [245].	It reduces obesity in mice by modulating the composition of the gut microbiota [246]. Its polysacarides were reported to increase plasma insulin levels and decrease plasma sugar levels in mice [247]. It is suggested to be used for the treatment of cardiovascular risk factors [248].	GL has decreased *Firmicutes*-to-*Bacteroidetes* ratios and reduced endotoxin-bearing *Proteobacteria* levels [246].
*Grifola frondosa* (Maitake mushroom)	Its water extract alleviates intestinal inflammation by suppressing TNF-α production and its signaling [249].	Antiobesity effects through the modulation of lipid metabolism via ceramide in mice fed a high-fat diet [250]. Its polysaccharides F2 and F3 improve insulin resistance in diabetic rats [251]. Positive effects of its heteropolysaccharide on NAFLD rats [252].	Andosan may also have influenced the composition and activity of microbiota in the A/J Min/+ mice [253].
*Hericium erinaceus* (Lion’s mane)	It suppressed the secretion of interleukin (IL)-6, interleukin (IL)-1β, tumor necrosis factor (TNF)-α and the expression of cyclooxygenase-2 (COX-2), inducible nitric oxide synthase (iNOS) in induced colitis mice [254].	Possesses anticancer, immuno-modulating, hypolipidemic, antioxidant and neuroprotective activities [255]. Improves mood and sleep disorders in patients affected by overweight or obesity [256]. Possesses antihyperglycemic and antihyperlipidemic activities in diabetic rats [257].	*Hericum erinaceus* renders changes in the composition and activity of the gastrointestinal tract microbiota that confer nutritional and health benefits to the host [255].
*Lentinula edodes* (Shiitake)	Its polysaccharides suppressed pro-inflammatory cytokines (tumor necrosis factor (TNF)-α, IL-6, IL-1β, and interferon (IFN)-γ) expression and colitis in mice [258].	Possesses immunomodulation, antioxidant, and antitumour effects [259]. β-Glucan from *Lentinula edodes* prevents cognitive impairments in high-fat diet-induced obese mice (involvement of colon–brain axis) [260]. Lentinula edodes-derived polysaccharide rejuvenates mice in terms of immune responses and gut microbiota [261].	L2 reverses the gut microbiota structure, such as the reduced *Firmicutes*-to-*Bacteroidetes* ratio, the increased *Bacteroidia*, the decreased *Bacilli* and *Betaproteobacteria*, the increased *Bacteroidaceae*, the decreased *Lactobacillaceae*, and *Alcaligenaceae* [262].

**Table 5:** Major foods that help leaky gut.

Foods	Major Intestinal Epithelial Regulation	Disease Model	Reference
Sucrose	Promoting intestinal cell proliferation and tumorigenesis	APC (Min) mice	Wang et al., 2009 [268]
Industrial orange by-products	Inflammatory cytokines and tight junction proteins	Acute colitis in mice	Pacheco et al., 2018 [269]
Peanut shell	Anti-inflammation	Acute colitis in mice	Lee et al., 2019 [270]
Sword bean	Inflammatory factors/NF-κB	Acute colitis in mice	Lee et al., 2019 [271]
Cinnamon	Suppresses IL-10	Chronic colitis in mice	Hagenlocher et al., 2017 [272]
Broccoli	Anti-inflammation	IBD in mice	Paturi et al., 2012 [273]
Red raspberries	Inflammatory factors/NF-κB	IBD in mice	Bibi et al., 2018 [274]
Blueberry	Inflammatory factors/NF-κB	IBD in mice	Pervin et al., 2016 [275]
Yogurt	Tight junction proteins	CaCo2 cells	Putt et al., 2017 [276]

## Data Availability

Not applicable.
